# Case report: Marked electroclinical improvement by fluoxetine treatment in a patient with *KCNT1*-related drug-resistant focal epilepsy

**DOI:** 10.3389/fncel.2024.1367838

**Published:** 2024-04-04

**Authors:** Ilaria Mosca, Elena Freri, Paolo Ambrosino, Giorgio Belperio, Tiziana Granata, Laura Canafoglia, Francesca Ragona, Roberta Solazzi, Ilaria Filareto, Barbara Castellotti, Giuliana Messina, Cinzia Gellera, Jacopo C. DiFrancesco, Maria Virginia Soldovieri, Maurizio Taglialatela

**Affiliations:** ^1^Department of Medicine and Health Sciences “Vincenzo Tiberio”, University of Molise, Campobasso, Italy; ^2^Department of Pediatric Neuroscience, Fondazione IRCCS Istituto Neurologico “Carlo Besta”, Milan, Italy; ^3^Department of Science and Technology, University of Sannio, Benevento, Italy; ^4^Department of Diagnostic and Technology, Fondazione IRCCS Istituto Neurologico “Carlo Besta”, Milan, Italy; ^5^Unit of Medical Genetics and Neurogenetics, Fondazione IRCCS Istituto Neurologico C. Besta, Milan, Italy; ^6^Department of Neurology, Fondazione IRCCS San Gerardo dei Tintori, Monza, Italy; ^7^Department of Neuroscience, University of Naples “Federico II”, Naples, Italy

**Keywords:** *KCNT1*, fluoxetine, next generation sequencing, drug repurposing, epilepsy

## Abstract

Variants in *KCNT1* are associated with a wide spectrum of epileptic phenotypes, including epilepsy of infancy with migrating focal seizures (EIMFS), non-EIMFS developmental and epileptic encephalopathies, autosomal dominant or sporadic sleep-related hypermotor epilepsy, and focal epilepsy. Here, we describe a girl affected by drug-resistant focal seizures, developmental delay and behavior disorders, caused by a novel, *de novo* heterozygous missense *KCNT1* variant (c.2809A > G, p.S937G). Functional characterization in transiently transfected Chinese Hamster Ovary (CHO) cells revealed a strong gain-of-function effect determined by the *KCNT1* p.S937G variant compared to wild-type, consisting in an increased maximal current density and a hyperpolarizing shift in current activation threshold. Exposure to the antidepressant drug fluoxetine inhibited currents expressed by both wild-type and mutant *KCNT1* channels. Treatment of the proband with fluoxetine led to a prolonged electroclinical amelioration, with disappearance of seizures and better EEG background organization, together with an improvement in behavior and mood. Altogether, these results suggest that, based on the proband’s genetic and functional characteristics, the antidepressant drug fluoxetine may be repurposed for the treatment of focal epilepsy caused by gain-of-function variants in *KCNT1*. Further studies are needed to verify whether this approach could be also applied to other phenotypes of the *KCNT1*-related epilepsies spectrum.

## Introduction

Variants in *KCNT1*, encoding for K_Na_1.1 subunits forming Na^+^-activated K^+^ channels, are associated with a wide spectrum of epileptic phenotypes, mainly including epilepsy of infancy with migrating focal seizures (EIMFS), developmental and epileptic encephalopathy (DEE) other than EIMFS, and autosomal dominant or sporadic sleep-related hypermotor epilepsy (Gertler et al., 2018; Bonardi et al., 2021). Moreover, *KCNT1* variants have been rarely reported in patients with focal epilepsy (Møller et al., 2015; Gertler et al., 2018).

*KCNT1*, similarly to highly-homologous KCNT2 (Cioclu et al., 2023) and classical voltage-gated Kv subunits, show a topological arrangement with six transmembrane segments (S_1_-S_6_) and both N- and C-termini located intracellularly. In particular, the C-terminus encompasses two regulators of K^+^ conductance (RCK1 and RCK2) domains located below the pore axis which form a large gating ring conferring channel opening sensitivity to intracellular Na^+^ ([Na]_i_); multiple binding sites for other cations and lipids have also been identified within this region (Zhang et al., 2023). Functional *KCNT1* channels assemble as homo- or heterotetramers and contribute to single action potential repolarization, single spike afterpotentials, or to the slow afterhyperpolarization (sAHP) which follows a burst of action potentials (Bonardi et al., 2021).

Irrespective of the clinical phenotype, the large majority of epilepsy-causing variants in *KCNT1* prompt gain-of-function (GoF) effects (Bonardi et al., 2021), thus suggesting that pharmacological strategies able to reduce channel function could be considered as precision-therapy approaches. Among them, *KCNT1* blockers such as the antiarrhythmic drug quinidine have been tested in few patients, although contrasting results regarding quinidine anticonvulsant efficacy have been reported; moreover, drug’s clinical use is further limited by concerns regarding its cardiac toxicity (Dilena et al., 2018; Fitzgerald et al., 2019; Liu et al., 2023).

In the present study, we report a patient with drug-resistant focal epilepsy carrying a novel, *de novo* missense *KCNT1* variant prompting GoF effects on channel function *in vitro*. We show that the anti-depressant fluoxetine counteracted mutation-induced functional defects *in vitro*, and that treatment with this drug resulted in a significant and long-lasting clinical improvement in the proband.

## Materials and methods

### Genetic analysis

Written informed consent was obtained for the patient and family members. Genomic DNA was extracted from peripheral blood lymphocytes as previously reported (Campostrini et al., 2018). The patient was investigated using a multigenic NGS panel (Agilent Sure Design, Santa Clara, CA) containing 300 genes (list available upon request) correlated with developmental epileptic encephalopathy (DEE). The average coverage at 20× for this panel was greater than 99%. The resulting sequences were aligned to the GRCh37/hg19 reference genome (MiSeq software). Data analysis was obtained using MiSeq Reporter vs. 2.4.60, Variant Studio vs. 2.2 (Illumina) and CLC Genomics Workbench vs. 7.0 (Qiagen). Variants with MAF > 1% were considered benign. Other variants were classified according to ACMG criteria (Richards et al., 2015). Parental segregation was performed by direct sequencing with Applied Biosystems (Life technologies) ABI 1330 XL automated sequencer.

### Mutagenesis and heterologous expression of channel subunits

The p.S937G variant herein investigated was engineered in a plasmid containing the cDNA for a myc-DDK tagged human isoform 2 (Q5JUK3–2) of *KCNT1* (RC214820; Origene, Rockville, MD, United States) by Quick-change mutagenesis, as previously described (Rizzo et al., 2016; Dilena et al., 2018). Mutant vector was verified by Sanger sequencing. Wild-type (WT) and mutant cDNAs were expressed in Chinese hamster ovary (CHO) cells by transient transfection using Lipofectamine 2000 (Invitrogen, Milan, Italy), as described (Rizzo et al., 2016; Dilena et al., 2018). A plasmid encoding for the Enhanced Green Fluorescent Protein (EGFP; Clontech, Palo Alto, CA) was used as a transfection marker. Total cDNA in the transfection mixture was kept constant at 4 μg.

### Electrophysiological recordings

Electrophysiological experiments were performed as previously described (Rizzo et al., 2016; Dilena et al., 2018). Briefly, macroscopic currents from transiently transfected CHO cells were recorded at room temperature (20°C-22°C) 24 h after transfection, with an Axopatch 200B amplifier (Molecular Devices, Union City, CA) using the whole-cell configuration of the patch-clamp technique. The pipette (intracellular) solution contained (in mM): 130 KCl, 10 NaCl, 10 HEPES, 5 EGTA, 5 Mg-ATP, pH 7.3–7.4 with HCl. Extracellular solution composition was (in mm): 138 NaCl, 5.4 KCl, 2 CaCl_2_, 1 MgCl_2_, 10 glucose, and 10 HEPES, pH 7.4 with NaOH. Data acquisition and analysis were performed as described (Rizzo et al., 2016; Dilena et al., 2018). To generate conductance-voltage curves, cells were held at −80 mV, then depolarized for 600 ms from −90 mV to +60 mV in 10-mV increments. Current densities (expressed in pA/pF) were calculated as peak K^+^ currents at all tested membrane potentials divided by cell capacitance (C). At each potential, peak currents (in pA) were subtracted from those measured in the previous pulse and normalized to the maximal current to obtain conductance values which were then fitted to a Boltzmann equation to obtain “V_½_” and “k” values. Drugs (Sigma-Aldrich, Milan, Italy) were dissolved in chloroform (quinidine; final vehicle concentration ≤ 0.01%) or DMSO (fluoxetine; final vehicle concentration ≤ 0.01%) and perfused using a fast solution exchange system as previously reported (Ambrosino et al., 2014). In these experiments, currents were activated by 1 s voltage ramps from −90 mV to +60 mV every 10 s; drug-induced current blockade was expressed as the percentage of +60 mV current inhibition produced by a 2 min drug application.

### Statistics

Each data point is the mean ± SEM of at least 7 determinations, each performed in different cells from at least 3 separate experiments. Statistically significant differences were evaluated with the Student’s t-test (indicated with *t*-test) or with the ANOVA followed by the Student–Newman–Keuls test (indicated with *ANOVA-SNK*), with the threshold set at *p* < 0.05.

## Results

### Case description and genetic analysis

The proband is a girl, now 20 years old, born after an uneventful pregnancy and delivery, without familiar history for epilepsy or other neurological diseases. Psychomotor development was normal during the first years of life.

**Figure 1:**
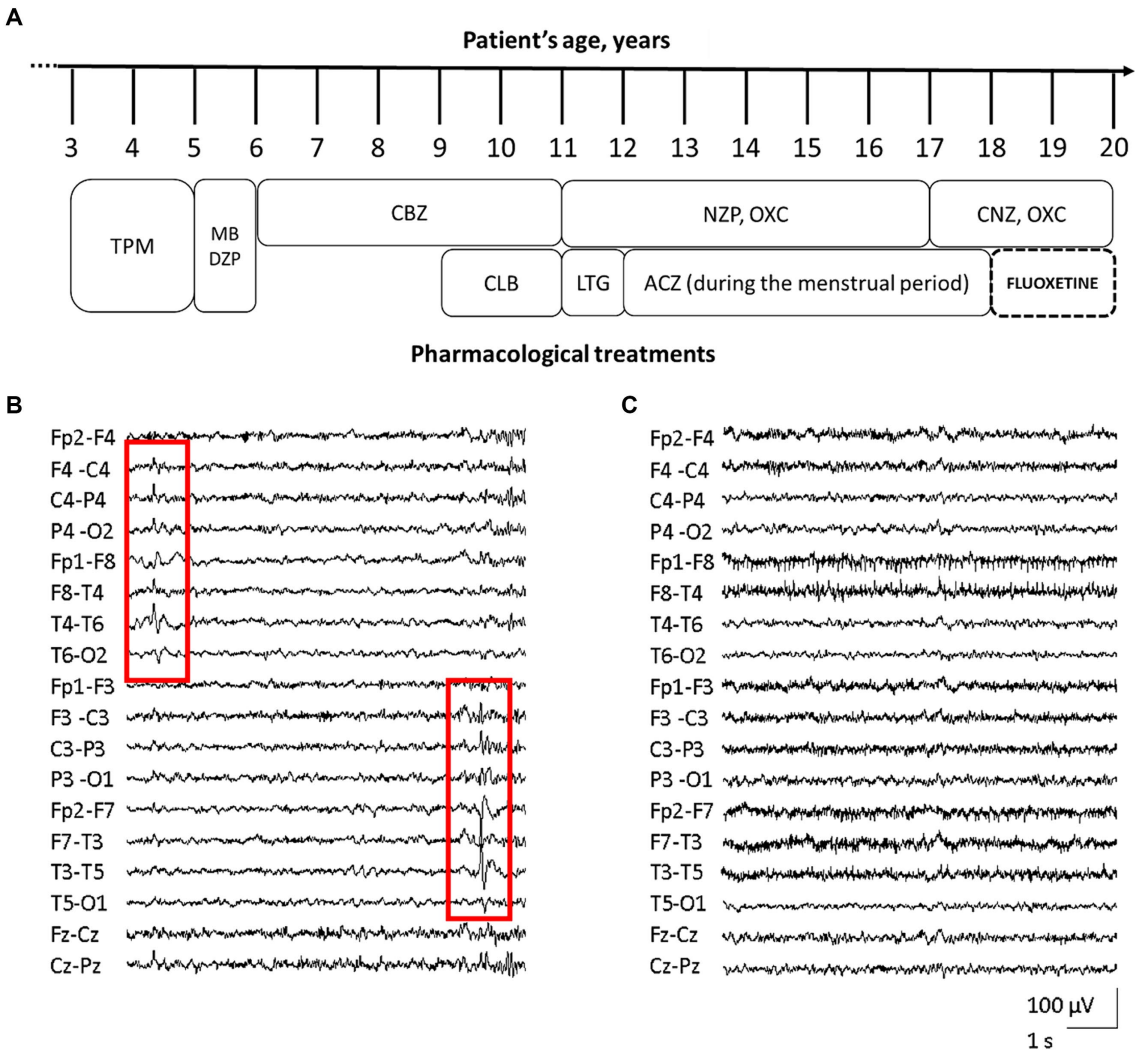
Patient’s pharmacological treatment, EEG traces before and after fluoxetine therapy. Schematic representation of the proband’s complex pharmacological treatment over time [legend of ASMs: acetazolamide (ACZ), carbamazepine (CBZ), clobazam (CLB), clonazepam (CNZ), diazepam (DZP), lamotrigine (LTG), methyl bromide (MB), nitrazepam (NZP), oxcarbazepine (OXC), topiramate (TPM)] **(A)**. EEG trace assessed before fluoxetine treatment, showing diffuse, low-amplitude, theta background activity, and bitemporal epileptiform abnormalities with alternating prevalence of side (red squares, **B**). EEG during treatment with fluoxetine, revealing the improvement of background activity with alpha-slow rhythm of increased amplitude, together with disappearance of epileptic activity **(C)**.

At the age of 3 years, she started presenting focal seizures with temporal semiology (staring, feeling of terror, swallowing, abdomen tightness). EEG reported epileptic spikes on the left temporal regions, while brain MRI was unremarkable (data not shown). Treatment with topiramate led to a transient resolution of seizures, but daily episodes recurred after few months. Topiramate was than replaced with methyl bromide and diazepam, however without benefit. In the next 3 years, reduction in seizures frequency from daily to monthly was achieved with carbamazepine and clobazam. At 11 y.o., the patient experienced focal *status epilepticus* (SE) interrupted by intravenous midazolam. Several antiseizure medications (ASMs) including lamotrigine, nitrazepam, oxcarbazepine, acetazolamide, and clonazepam, resulted ineffective (Figure 1A). With menarche at the age of 13 y.o., seizures frequency increased during menses.

The patient came to our attention at 16 years of age. EEG showed diffuse, low-amplitude, theta background activity, and bitemporal epileptiform abnormalities with alternating prevalence of side (Figure 1B). Her neurological examination was characterized by severe intellectual disability (IQ 44), with anxiety disorder and aggressiveness. At that time the girl was experiencing daily seizures, despite treatment with oxcarbazepine and nitrazepam. Repeated brain MRI and CGH-array were unrevealing. NGS target panel for epilepsy genes detected the novel heterozygous c.2809A > G (NM_020822.2) missense variant in *KCNT1*, causing the p.S937G substitution (rs1554778379), resulting *de novo* in the proband.

### Functional properties of wild-type and p.S937G mutant *KCNT1* channels

**Figure 2:**
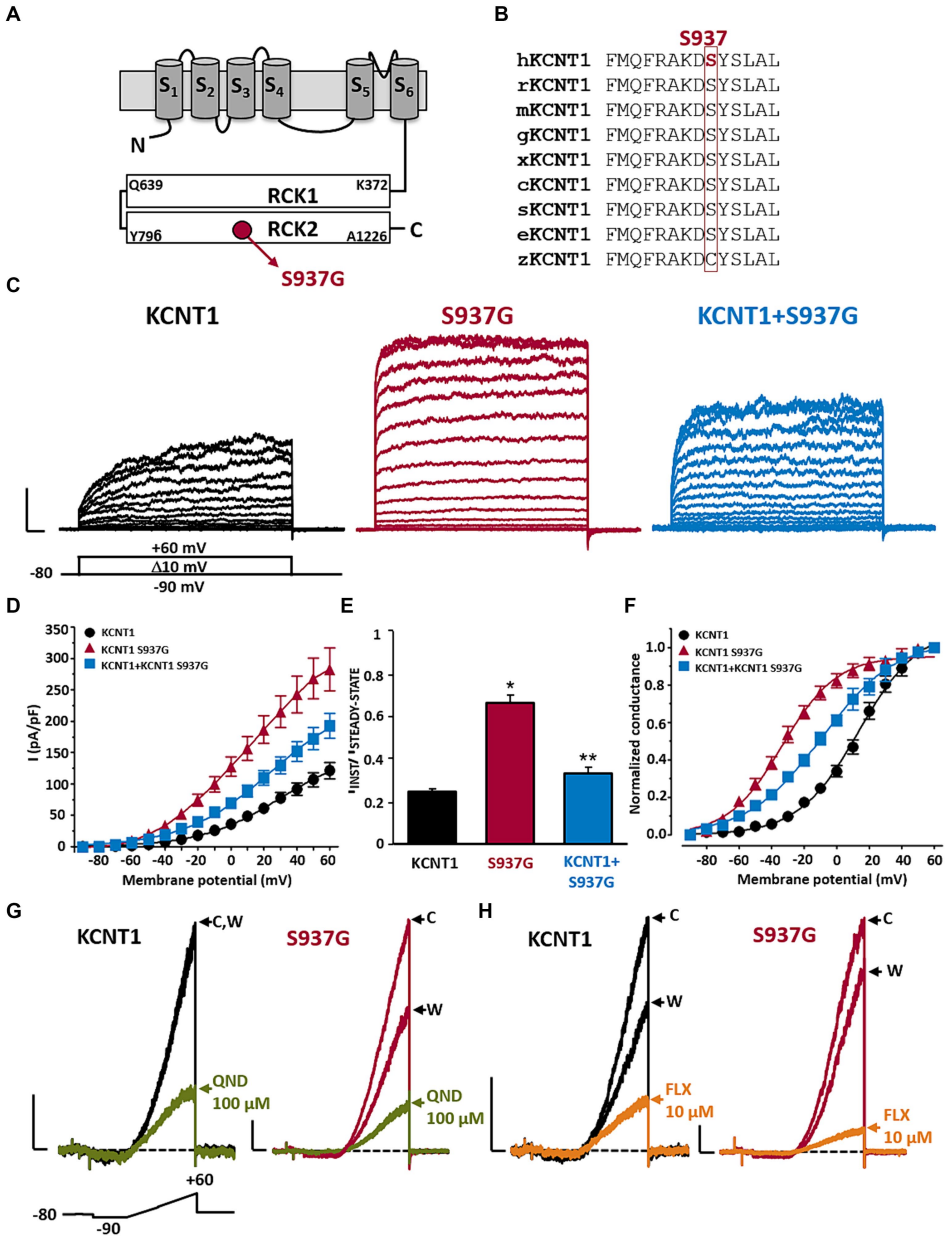
Functional and pharmacological characterization of *KCNT1* and *KCNT1* p.S937G channels. Topological representation of a *KCNT1* subunit and localization of the p.S937G variant **(A)**. Partial alignment of *KCNT1* protein among species **(B)**. Representative family traces recorded in cells expressing the indicated channels in response to the voltage protocol shown below the leftmost traces **(C)**. Current scale: 1 nA; time scale: 50 ms. Average of I_INST_/I_STEADY-STATE_ ratios **(D)**, current densities **(E)**, and conductance/voltage curves **(F)** measured in cells expressing the indicated channels. Representative traces recorded in response to the ramp protocol shown below the leftmost traces in cells expressing the indicated channels in control solution (indicated with C), after 2-min exposure to 100 μM quinidine (QND; blue traces in panel **G**) or 10 μM fluoxetine (FLX; green traces in panel **H**), or upon drug washout (indicated with W). Current scale: 500 pA; time scale: 200 ms. ^*^ = *p* < 0.05 versus *KCNT1*; ^**^ = *p* < 0.05 versus *KCNT1* + S937G.

The *KCNT1* p.S937G variant is localized in the RCK2 domain (Figure 2A), affecting a residue highly conserved among species (Figure 2B). To investigate the functional properties of *KCNT1* channels incorporating this novel variant, we performed *in vitro* experiments in CHO cells expressing WT or mutant *KCNT1* subunits. As previously reported (Rizzo et al., 2016; Dilena et al., 2018), transfection with *KCNT1* cDNA elicited outwardly-rectifying currents in response to depolarizing voltage pulses from −90 mV to +60 mV (maximal current density at +60 mV was 121.1 ± 12.9 pA/pF; *n* = 34; Figures 2C,D). *KCNT1* currents displayed complex activation kinetics, with an instantaneous, time-independent component (I_INST_), followed by a slower, time-dependent one (I_STEADY-STATE_ – I_INST_). At +60 mV, the ratio between currents measured at the beginning (I_INST_) and at the end (I_STEADY-STATE_) of the depolarizing step was 0.25 ± 0.01 (*n* = 46; Figure 2E). Expression of homomeric *KCNT1* p.S937G channels also generated outwardly-rectifying currents. When compared to WT, current densities of mutant *KCNT1* at +60 mV were significantly larger (maximal current density at +60 mV was 282.6 ± 34.0 pA/pF; *n* = 25; *p* < 0.05 versus WT, *t*-test; Figures 2C,D), and the I_INST_/I_STEADY-STATE_ ratio was significantly increased (I_INST_/I_STEADY-STATE_ was 0.67 ± 0.04; *n* = 23; *p* < 0.05 versus WT, *t*-test; Figures 2C–E). Moreover, the G/V curve was shifted in the hyperpolarizing direction (Figure 2F); indeed, Boltzmann analysis of the G/V for *KCNT1* p.S937G channels revealed that the activation midpoint (V_1/2_) was significantly more negative when compared to WT channels (V_1/2_ were 13.6 ± 2.1 mV or − 33.4 ± 1.6 mV for *KCNT1* or *KCNT1* p.S937G channels, respectively; n = 19 or 25, respectively; *p* < 0.05, *t*-test). By contrast, no significant difference was measured in the slope (*k* values were 18.7 ± 1.1 or 16.7 ± 1.1 mV/*e*fold for *KCNT1* or *KCNT1* p.S937G channels, respectively; *p* > 0.05, *t*-test). Qualitatively similar, although quantitatively smaller, effects were measured upon co-expression of mutant *KCNT1* p.S937G with WT subunits, an experimental strategy mimicking the heterozygous condition of the proband carrying the variant on a single allele. The maximal current density at +60 mV was 192.2 ± 19.7 pA/pF (Figure 2D), the I_INST_/I_STEADY-STATE_ ratio was 0.34 ± 0.03 (Figure 2E), and the V_1/2_ was −9.1 ± 2.2 mV (Figure 2F; *n* = 22–23; *p* < 0.05 versus *KCNT1* and *KCNT1* p.S937G homomers, *ANOVA-SNK*).

Taken together, these results suggest that the *KCNT1* p.S937G variant leads to strong GoF effects by increasing the amplitude of the maximal currents and shifting their activation threshold toward hyperpolarized potentials.

### Pharmacological modulation of wild-type and p.S937G mutant *KCNT1* channels

To counteract the mutation-induced GoF changes, we tested *in vitro* the effect of different KCNT blockers, including the antiarrhythmic drug quinidine (QND). As previously demonstrated (Rizzo et al., 2016; Dilena et al., 2018), 100 μM QND inhibited WT currents at +60 mV by 70.2 ± 4.1% (*n* = 7; Figure 2G); *KCNT1* current blockade by QND was fully reversible after ~3 min of drug washout (Figure 2G). A slight increase in current inhibition was measured in *KCNT1* p.S937G currents (% of current inhibition at +60 mV was 81.6 ± 2.4, *n* = 12; *p* < 0.05 versus WT, *t*-test; Figure 2G), suggesting a possible therapeutic use of this drug. However, considering the poor compliance to quinidine treatment, in view of the scarce clinical efficacy and its potential cardiac toxicity (Dilena et al., 2018; Fitzgerald et al., 2019; Liu et al., 2023), we searched for alternative approaches. Among KCNT blockers, we focused on the well-known antidepressant drug fluoxetine (FLX), since we recently showed that this drug was effective in blocking currents expressed by homologous KCNT2 channels (both WT and carrying DEE-causing GoF pathogenic variants) (Cioclu et al., 2023).

Exposure to 10 μM FLX blocked 79.8 ± 2.0% of *KCNT1* currents elicited at +60 mV (*n* = 14; Figure 2H); currents largely recovered after ~3 min of drug washout (Figure 2H). FLX was slightly more effective in blocking *KCNT1* p.S937G currents (% of current inhibition at +60 mV was 86.7 ± 1.7%; *n* = 9; *p* < 0.05 versus WT, t-test; Figure 2H). Similar results were observed on *KCNT1* + *KCNT1* p.S937G heteromeric channels, showing a current reduction of 87.4 ± 1.7% (*n* = 14; *p* < 0.05 versus WT, *p* > 0.05 versus *KCNT1* p.S937G, ANOVA-SNK; Figure 2H).

### Precision approach with fluoxetine

Considering the presence of an active anxiety disorder, a clear indication to FLX therapy, seizures not responding to several ASMs, and according to the described *in vitro* data showing the ability of FLX to block currents expressed by p.S937G *KCNT1* channels, at the age of 18 years the patient started treatment with this drug. With an initial dose of 10 mg per day, the posology was gradually increased until 60 mg/day (1.2 mg/kg), with a plasma level of 412.6 ng/mL, resulting well tolerated.

During FLX treatment, the patient reported a clear and prolonged clinical benefit with disappearance of seizures, except for sporadic episodes during a SARS-COV-2 infection. EEG recording revealed alpha-slow background activity of greater amplitude, together with disappearance of epileptic abnormalities (Figure 1C).

After 2 years of follow-up, the patient is still under treatment with fluoxetine. Due to the absence of further seizures, acetazolamide was deemed no longer necessary, while the other ASMs were not reduced as requested by the patient and her parents. Along with amelioration of the epileptic features, the patient also reported a significant improvement in behavior and mood, and the achievement of important school milestones with high school graduation.

## Discussion

In the present study we report a patient with drug-resistant focal epilepsy, developmental delay and behavior disorders carrying a novel *KCNT1 de novo* heterozygous missense variant (p.S937G). This variant affects a highly conserved amino acid residue located in the regulator of K^+^ conductance-2 (RCK2) domain (Zhang et al., 2023), a critical region for channel gating. Indeed, similarly to the largest majority of previously-described *KCNT1* pathogenic variants (Rizzo et al., 2016; Dilena et al., 2018; Gertler et al., 2018), mutant channels displayed significant GoF effects in terms of increased current density and leftward shift in the voltage-dependence of activation, both in homomeric and heteromeric channels when expressed together with wild-type subunits. In addition to *KCNT1*, GoF variants in other potassium channel genes, including KCNQ2 and KCNQ3 (Miceli et al., 2015; Sands et al., 2019), KCNT2 (Cioclu et al., 2023), among several others (Niday and Tzingounis, 2018), are associated with hyperexcitability-linked neurological diseases; how GoF variants in these channels affect neuronal and networks function is largely unknown. Among potential mechanisms, it has been hypothesized that *KCNT1* GoF variants lead to network hyperexcitability by: I- selectively accelerating action potential repolarization in excitatory neurons, thereby limiting sodium channel inactivation and causing a higher spiking frequency; II- selectively reducing membrane excitability or inducing spike frequency adaptation in inhibitory neurons, resulting in disinhibition; or III- promoting developmental alterations in synaptic connectivity.

In the cortex and hippocampus, single-cell RNA sequencing data suggest that *KCNT1* is robustly expressed in parvalbumin-positive (PV+) and somatostatin-positive (SOM+) interneuron subsets, almost absent in vasoactive intestinal peptide-positive (VIP+) interneurons, while it shows intermediate expression in excitatory pyramidal neurons (Cembrowski et al., 2016; Erö et al., 2018). Notably, recent results from animal models in which *KCNT1* GoF variants have been introduced suggest that preferential dampening of intrinsic excitability in specific subsets of cortical (Shore et al., 2020) and hippocampal (Gertler et al., 2022) inhibitory neurons provides a major contribution to network hyperexcitability and hypersynchronization, leading to changes in synaptic connectivity, disrupted excitation/inhibition balance, and seizures (Shore et al., 2020).

In the case here reported, despite treatment with different ASMs, focal seizures persisted at high frequency; thus, we searched for pharmacological approaches to counteract the increase of *KCNT1* currents caused by the novel p.S937G variant. Thus, we first tested and showed the ability of the KCNT blocker quinidine to reduce *in vitro* GoF effects caused by the variant. However, due to the unsatisfactory clinical response and to well-known cardiac concerns associated to quinidine treatment (Dilena et al., 2018; Cioclu et al., 2023; Zhang et al., 2023), and considering that patients with the best quinidine response have variants localized distal to the RCK2 domain (Fitzgerald et al., 2019), we searched for an alternative compound with potentially better efficacy and safety profiles.

Fluoxetine, a well-tolerated anti-depressant drug, has been suggested as a repurposed precision therapy for severe epilepsies caused by GoF variants in other neuronal Kv channels (Ambrosino et al., 2023; Cioclu et al., 2023). Thus, we tested *in vitro* the effect of fluoxetine on *KCNT1* p.S937G channels, demonstrating that the drug’s ability to counteract the GoF features observed in channels incorporating the p.S937G variant. Thus, considering the behavioral disturbances (anxiety disorders, aggressiveness) manifested by the proband, and considering the scarce efficacy of several other ASMs, she started treatment with fluoxetine, resulting in a prolonged clinical and electroencephalographic improvement, with a complete seizure disappearance and a marked improvement in mood and behavior.

The clinical response to fluoxetine treatment observed in the herein reported transitional age patient cannot be accounted for by the disease natural history, but seems to be correlated to the drug inclusion in the therapeutic regimen. At steady-state, fluoxetine plasma level resulted within the range observed during antidepressant treatment (120–500 ng/mL), achieving a drug concentration range of 0.6–2 μM, similar to those prompting a significant block of *KCNT1* currents *in vitro*. Being highly lipophilic, fluoxetine can reach elevated concentrations in the brain (Bolo, 2000) (brain/plasma ratio is about 10), suggesting that the block of *KCNT1* current observed with this drug may have contributed to the clinical amelioration observed in the proband. However, it should be highlighted that fluoxetine cannot be considered a *KCNT1*-specific blocker, since this drug also blocks other potassium channels, such as KCNT2 (Cioclu et al., 2023), Kv3.1 (Choi et al., 2001) and Kv1.3 (Choi et al., 1999), with similar potency; thus, a direct causal relationship between drug-dependent blockade of *KCNT1* currents and amelioration of the underlying phenotype is difficult to establish. Moreover, the occurrence of disease-related compensatory mechanisms (Pan et al., 2018), possibly including the up-regulation of additional ion channel targets contributing to treatment efficacy (Hill et al., 2022), should also be taken into account. Nonetheless, given that fluoxetine is widely-prescribed for the treatment of major depressive disorder and obsessive-compulsive disorder in pediatric patients, we hope that our results might be of help not only for adults, but also for pediatric patients with *KCNT1*-related disorders unresponsive to conventional AED treatment.

## Conclusion

We herein report a gene-based approach with fluoxetine in a patient with drug-resistant focal epilepsy due to a novel *KCNT1* GoF variant. In this case, fluoxetine treatment, following several “conventional” ASMs that resulted ineffective, led to a complete control of seizures. Repurposing drugs selected on the basis of probands’ genetic and functional characteristics for the treatment of severe forms of epilepsy represents one of the main strategies for a precision medicine therapy (Sisodiya, 2021). Further studies are needed to verify whether this approach could be considered also for other phenotypes of the *KCNT1*-related epilepsy spectrum, including EIMFS.

## Data availability statement

The data presented in the study are deposited in the Zenodo repository, accession number 10.5281/zenodo.10623783.

## Ethics statement

The studies involving humans were approved by Fondazione IRCCS Istituto Neurologico “Carlo Besta”, Milan. The studies were conducted in accordance with the local legislation and institutional requirements. Written informed consent for participation in this study was provided by the participants' legal guardians/next of kin. The study was conducted in accordance with the local legislation and institutional requirements. Written informed consent was obtained from the individual(s) for the publication of any potentially identifiable images or data included in this article.

## Author contributions

IM: Investigation, Writing – review & editing. EF: Investigation, Writing – review & editing. PA: Investigation, Supervision, Writing – review & editing. GB: Investigation, Writing – review & editing. TG: Conceptualization, Supervision, Writing – review & editing. LC: Data curation, Supervision, Writing – review & editing. FR: Investigation, Writing – review & editing. RS: Investigation, Writing – review & editing. IF: Investigation, Writing – review & editing. BC: Investigation, Methodology, Writing – review & editing. GM: Investigation, Writing – review & editing. CG: Supervision, Writing – review & editing. JCD: Conceptualization, Writing – original draft, Writing – review & editing. MVS: Conceptualization, Data curation, Investigation, Methodology, Supervision, Writing – original draft, Writing – review & editing. MT: Conceptualization, Investigation, Supervision, Writing – original draft, Writing – review & editing.
